# Downregulation of RAB7 and Caveolin-1 increases MMP-2 activity in renal tubular epithelial cells under hypoxic conditions

**DOI:** 10.1515/med-2021-0341

**Published:** 2021-09-29

**Authors:** Wenmin Yu, Xiumei Ke, Meiren Li, Ping Ye, Jing Peng, Huimin Li

**Affiliations:** The School of Basic Medical Science, Jiujiang University, 320 XunYang East Road, Jiujiang 332000, Jiangxi, People’s Republic of China; Jiujiang University Clinic College/Hospital, Jiujiang University, Jiangxi, People’s Republic of China

**Keywords:** proximal tubular cells, hypoxia, autophagy, endocytosis, matrix metalloproteinase-2

## Abstract

Tubulointerstitial fibrosis leads to tubular basement membrane thickening and accumulation of interstitial extracellular matrix (ECM). Matrix metallopeptidase-2 (MMP-2) is involved in the breakdown of ECM. Chronic hypoxia often occurs in the kidney tissues of patients with chronic kidney disease. Our previous study reported the effect of autophagy and endocytosis on MMP-2 activity in hypoxia-treated HK-2 cells. In this study, the relationship between the Ras-related protein Rab-7a (RAB7) and MMP-2 activity was further investigated. RAB7 overexpression decreased MMP-2 activity. In contrast, the results for RAB7 knockdown displayed the opposite pattern. Short hairpin RNA technology was used to knockdown Caveolin-1 (Cav-1) or Beclin-1 (Bec-1) in HK-2 cells. The two genes displayed differential effects on MMP-2 activity. Cav-1 and RAB7 interference increased MMP-2 activity. This study suggested that autophagy and endocytosis, RAB7, Cav-1, and Bec-1 may serve as potential mediators for altered MMP-2 activity.

## 1 Introduction

Renal tubulointerstitial fibrosis is the pathological feature of almost all chronic kidney diseases (CKDs) [1]. Tubulointerstitial fibrosis leads to the thickening of the basement membrane of tubules and interstitial extracellular matrix (ECM) accumulation [2,3]. Matrix metallopeptidase-2 (MMP-2) participates in ECM destruction and degrades type IV collagen (Col-IV), the main component of the basement membrane [4,5,6]. In patients with CKD, MMP-2 activity in renal tissues decreases, leading to progressive renal insufficiency and organ failure [7]. However, the underlying mechanism is not completely understood. Chronic hypoxia often occurs in the kidney tissues of patients with CKD. *In vitro* data suggests that hypoxia can induce functional and phenotypic changes in renal epithelial cells and fibroblasts [8]. For example, MMP-2 expression and activity in hepatic stellate cells are reduced under hypoxic conditions [9]. Numerous studies have reported that the activity of MMP-2 in proximal tubule cells decreases during hypoxia, but the underlying mechanism remains unclear [10]. We also had reported earlier that hypoxia decreases MMP-2 activity [11,12]. Recent studies have demonstrated that autophagy has a close and complex relationship with hypoxia, but the underlying mechanism was not reported earlier [13]. In addition, researchers have reported that autophagy serves a dual role in hypoxia-induced cell damage [14]. Hypoxia, a cellular adaptive response, reduces Na/K-ATPase activity in the alveolar epithelium by activating the endocytosis of the α1 subunit [8,15]. The aforementioned studies indicated that hypoxia induces autophagy and endocytosis. Ras-related protein Rab-7a (RAB7) participates in the final stage of autophagy [16], and some studies have reported that RAB7 is involved in the autophagic process [17]. Moreover, the role of RAB7 in autophagosome lysosome fusion has also been confirmed [18]. RAB7 indicates the maturation of endosomes and autophagosomes. Thus RAB7 serves as an effective multifunctional regulatory factor for autophagy and endocytosis [19,20,21]. In summary, RAB7 serves an important role in the maturation of autophagosomes and endosomes. Recently, a study demonstrated that the relationship between RAB7 and MMP-2 activity occurred in a dependent manner, which protected against albumin-induced injury [22]. RAB7 is a key molecule of autophagy and endocytosis [19]. If the effect of RAB7 on MMP-2 can be determined, the relationship between autophagy, endocytosis, and MMP-2 will be further confirmed at the molecular level. The role of RAB7 in MMP-2 in renal tubular epithelial cells with hypoxia-induced injury is not completely understood.

Furthermore, our previous study investigated the effects of autophagy and endocytosis on MMP-2 activity in human renal proximal tubular cells under hypoxic conditions [12,23]. To further investigate the possible effects of autophagy and endocytosis on the activity of MMP-2 at the molecular level in HK-2 cells under hypoxic conditions, this study also investigated the effects of Beclin-1 (Bec-1) and Caveolin-1 (Cav-1) and MMP-2 activity in the culture medium of hypoxia-treated HK-2 cells *in vitro*. As Bec-1 and Cav-1 are key genes for autophagy and endocytosis, respectively [24,25], if the effects of Cav-1 and Bec-1 on MMP-2 activity can be determined, autophagy and endocytosis on MMP-2 activity can be further determined, and it is also possible to confirm whether autophagy or endocytosis has any effect on MMP-2 activity. Proximal tubular cells have a key role in the development of renal fibrosis [11]. Therefore, the results of this study may aid the development of therapeutic strategies to control the progression of renal fibrosis.

## 2 Materials and methods

### 2.1 Cell culture

The human renal proximal tubular epithelial cell line (HK-2) was purchased from the Cell Bank of Type Culture Collection of the Chinese Academy of Sciences. HK-2 cells were cultured in DMEM (high-glucose; Thermo Fisher Scientific, Inc.) supplemented with 10% FBS, 100 units per mL penicillin, and 100 μg/mL streptomycin (Thermo Fisher Scientific, Inc.).

Cells (5 × 10^5^ cells per well) were seeded into 6-well plates and cultured for 24 h. To simulate hypoxic conditions, cells were cultured under hypoxic conditions, which were maintained using a compact gas oxygen controller (Thermo Fisher Scientific, Inc.), and held under positive pressure in an atmosphere of 92–94% N_2_, 5% CO_2_, and 1–3% O_2_ for 24 h. Control cells were cultured in normoxic conditions [12].

### 2.2 Stable transfection of cells

The green fluorescent protein (GFP)-RAB7 (RAB7 gene No. HSU44104) lentiviral vector was purchased from Shanghai GenePharma Co., Ltd. Cells were transfected with the GFP-RAB7 lentiviral vector according to the manufacturer’s protocol. Short hairpin (sh)RNAs targeted against RAB7 (GGTTATCATCCTGGGAGATTCTGGA) were delivered into HK-2 cells using a lentiviral vector (Hanbio Biotechnology Co., Ltd.) according to the manufacturer’s instructions. shRNAs targeted against Bec-1 (CTCAAGTTCATGCTGACGAAT; Hanbio Biotechnology Co., Ltd.) and Cav-1 (GCTTTGTGATTCAATGTCTAA; Hanbio Biotechnology Co., Ltd.) were delivered into HK-2 cells using a lentiviral vector (Hanbio Biotechnology Co., Ltd.) according to the manufacturer’s protocol. G418 (500 μg/mL) was used for the selection of stably transfected cells.

### 2.3 Histopathology and immunohistochemistry

Renal tissues were fixed in 4% paraformaldehyde and the sections were embedded in paraformaldehyde. The renal biopsy specimens of six cases were stained with hematoxylin and eosin (HE), which was used to identify alterations in the renal structure. RAB7 and hypoxia-inducible factor 1-α (HIF-1α) expression levels were detected following xylene dewaxing of the sections and dehydration using a graded ethanol series. Endogenous peroxidase activity was blocked by incubating the slides with 0.3% H_2_O_2_ for 5 min. Following washing three times with PBS, the slides were incubated with primary RAB7 and HIF-1α (1:200; ProteinTech China) antibodies overnight at 4°C. Following washing with PBS, the slides were then incubated with an HRP-conjugated secondary antibody (1:200; ProteinTech China) at 37°C for 1 h. Diaminobenzidine (Olympus Corporation) was used to visualize the sections, and nuclei were counterstained with hematoxylin.

### 2.4 Western blotting

Total protein was extracted from the cells using an immunoprecipitation assay buffer containing protease inhibitors (Vazyme Biotech Co., Ltd.). Proteins (40 µg) were separated via sodium dodecyl sulfate-polyacrylamide gel electrophoresis (SDS-PAGE) and transferred to polyvinylidene fluoride membranes. The membranes were blocked with 5% non-fat milk in TBST (0.5% Triton X-100) for 1 h at room temperature. Subsequently, the membranes were incubated overnight at 4°C with primary antibodies targeted against RAB7 (Abcam), Bec-1 (Abcam), Cav-1 (Abcam), and GAPDH (ProteinTech China). The membranes were washed three times with TBST and then incubated at room temperature with a horseradish peroxidase-conjugated secondary antibody (ProteinTech China) for 1 h. Protein bands were visualized using enhanced chemiluminescence (Vazyme Biotech Co., Ltd.) and CL-XPosure Film (Thermo Fisher Scientific, Inc.) [23].

### 2.5 ELISA to quantify MMP-2 and Col-IV in culture media

The contents of MMP-2 and Col-IV protein in the culture media were measured using ELISA kits (Shanghai Hengyuan Biological Technology Co., Ltd.) according to the manufacturer’s instructions. Purified MMP-2 or Col-IV antibodies were applied to coat the microdroplet plate. Subsequently, culture media was added and incubated at 37˚C for 2 h. After washing, HRP-conjugated MMP-2 or Col-IV antibodies were added. The plate was thoroughly washed and then 3,3′,5,5′-tetramethylbenzidine substrate was added. Sulfuric acid solution was added to stop the reaction, and the color was determined via spectrophotometry at a wavelength of 450 nm. MMP-2 and Col-IV protein contents in the culture media were calculated according to the standard curve [12].

### 2.6 Detection of MMP-2 activity by zymography

MMP-2 activity was detected using the Zymography Assay kit (Applygen Technologies, Inc.) according to the manufacturer’s protocol. MMP-2 protein was separated via SDS-PAGE. Subsequently, SDS was extracted from the gel using Triton X-100 (0.1%) and incubated for 48 h at 37°C. The gels were stained with coomassie brilliant blue G250 and decolorized. The bright band against the blue background indicated MMP-2 activity. Image Master 1D Analysis software (Pharmacia Biotech) was used to identify the bright band [12].

### 2.7 Statistical analysis

Statistical analyses were performed using SPSS software (version 13.0; SPSS, Inc.). Data are presented as mean ± SD. The Student *t*-test (unpaired *t*-test) was used to analyze the differences among groups. *P* < 0.05 was considered to indicate a statistically significant difference.

**Ethics statement:** Six human renal biopsy specimens were obtained with informed consent from the patients of Jiujiang University Subsidiary Hospital. We ensured that the participants’ rights were protected. In this study, all manipulations were approved by the Medical Ethics Committee of Jiujiang (Approved ID: 1–2013, 20 February 2013).

## 3 Results

### 3.1 Verification of RAB7 overexpression and knockdown in HK-2 cells

**Figure 1:**
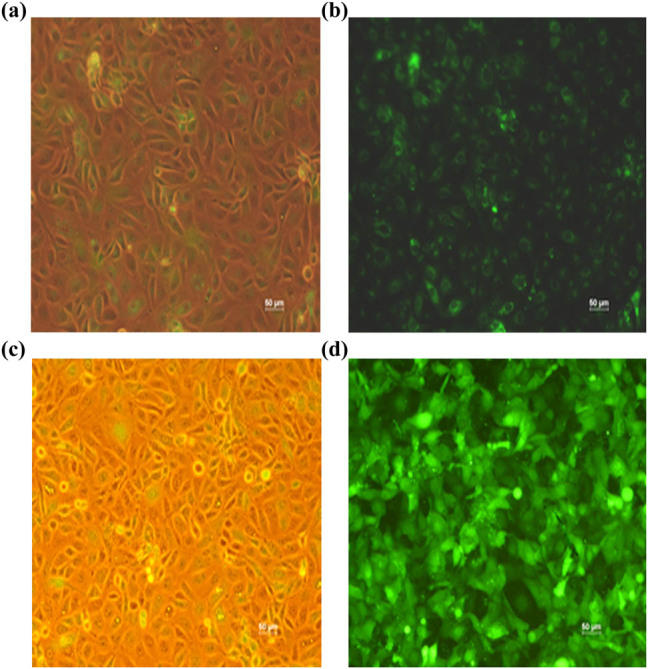
Stable infection of HK-2 cells. (a) Hk-2 cells under no fluorescence microscope. (b) The GFP-RAB7 lentiviral vector was infected into HK-2 cells under a fluorescence microscope. The success rate of infection was almost accounted for 100%. RAB7 protein was often expressed in cytoplasm, and the results showed that the position of RAB7 protein is correct. (c) HK-2 cells under no fluorescence microscope. (d) shRNA lentiviral vector directed toward RAB7 were delivered HK-2 cells under fluorescence microscope. The success rate of infection was almost accounted for 100%.

To verify the effect of RAB7 on MMP-2 activity in HK-2 cells under hypoxic conditions, a stable HK-2 cell line overexpressing RAB7 was established. The GFP-RAB7 lentiviral vector was infected into HK-2 cells under a fluorescence microscope. The success rate of infection can reach almost 100% after G418 (500 μg/mL) was used for the selection of stably transfected cells (Figure 1a and b). A stable HK-2 cell line infected with RAB7 shRNA was also established, which displayed an infection rate of 100% after G418 (500 μg/mL) was used for the selection of stably transfected cells (Figure 1c and d).

### 3.2 RAB7 protein expression levels in human renal biopsy specimens and GFP-RAB7-transfected HK-2 cells under hypoxic conditions detected by histopathology and immunostaining

**Figure 2:**
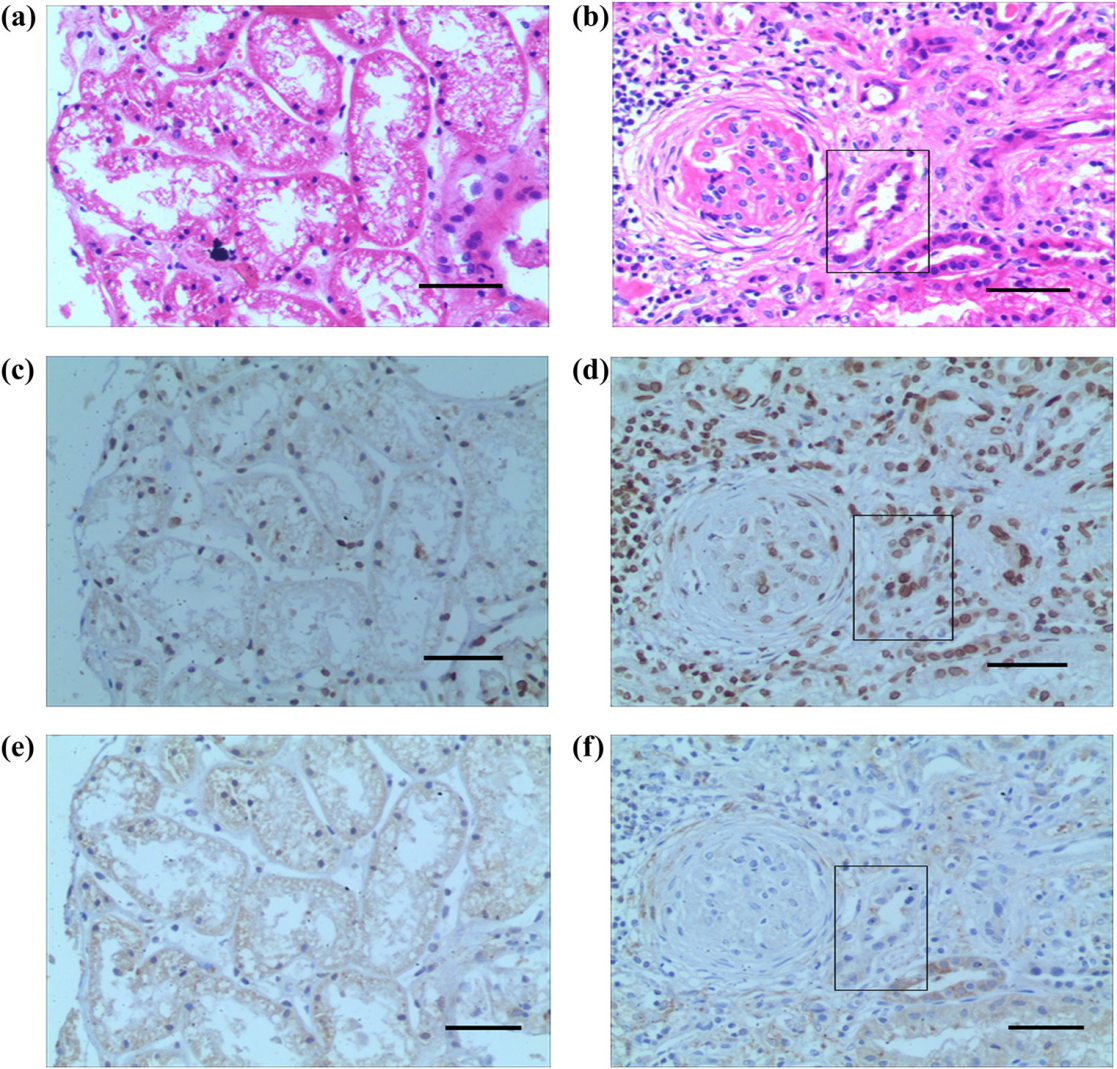
Expression of RAB7 protein in human renal biopsy specimens. (a) HE staining was used where there was no fibrosis. (b) The expression of HIF-1α was detected where there was no fibrosis. (c) The expression of RAB7 was detected where there was no fibrosis. (d) HE staining was used in the fibrotic area (displayed in the box). (e) The expression of HIF-1α was significant in the fibrotic area (displayed in the box). (f) The expression of RAB7 was not affected by fibrosis (displayed in the box).

**Figure 3:**
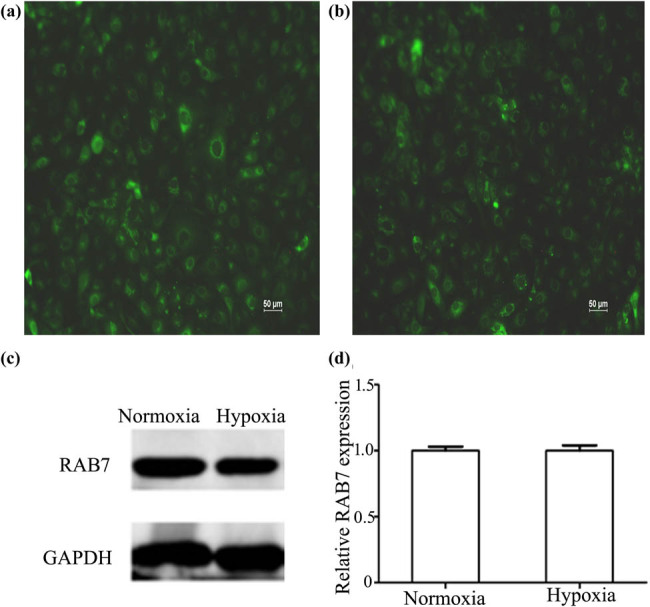
Expression of RAB7 protein in HK-2 cells with GFP-RAB7 lentiviral vector under hypoxia or normoxia. (a) Expression of RAB7 protein in HK-2 cells with GFP-RAB7 lentiviral vector under normoxia. (b) Expression of RAB7 protein in HK-2 cells with GFP-RAB7 lentiviral vector under hypoxia. The results show no difference. (c) Expression of RAB7 protein in HK-2 cells with GFP-RAB7 lentiviral vector under hypoxia and normoxia was detected by western blot. The results show no significant difference. (d) Expression of RAB7 relative to that of GAPDH. Error bars represented mean ± SD, and the results were analyzed by unpaired with Student’s *t*-test, *p < 0.05, **p < 0.01.

A total of six human renal biopsy specimens (nephrotic syndrome; CKD) were subjected to HE staining. In the fibrotic area, the expression of HIF-1α was significantly enhanced, whereas in non-fibrotic areas, the expression of HIF-1α was significantly reduced. By contrast, the expression of RAB7 was not affected by fibrosis (Figure 2f). To further detect the protein expression levels of RAB7 in HK-2 under hypoxic conditions, the RAB7 protein expression levels in GFP-RAB7-infected HK-2 cells under hypoxic and normoxic conditions were determined via western blotting. The results indicated that the RAB7 protein expression level was not altered (Figure 3a–d).

### 3.3 Effect of RAB7 overexpression and knockdown on MMP-2 activity in the culture medium of HK-2 cells under hypoxic conditions

**Figure 4:**
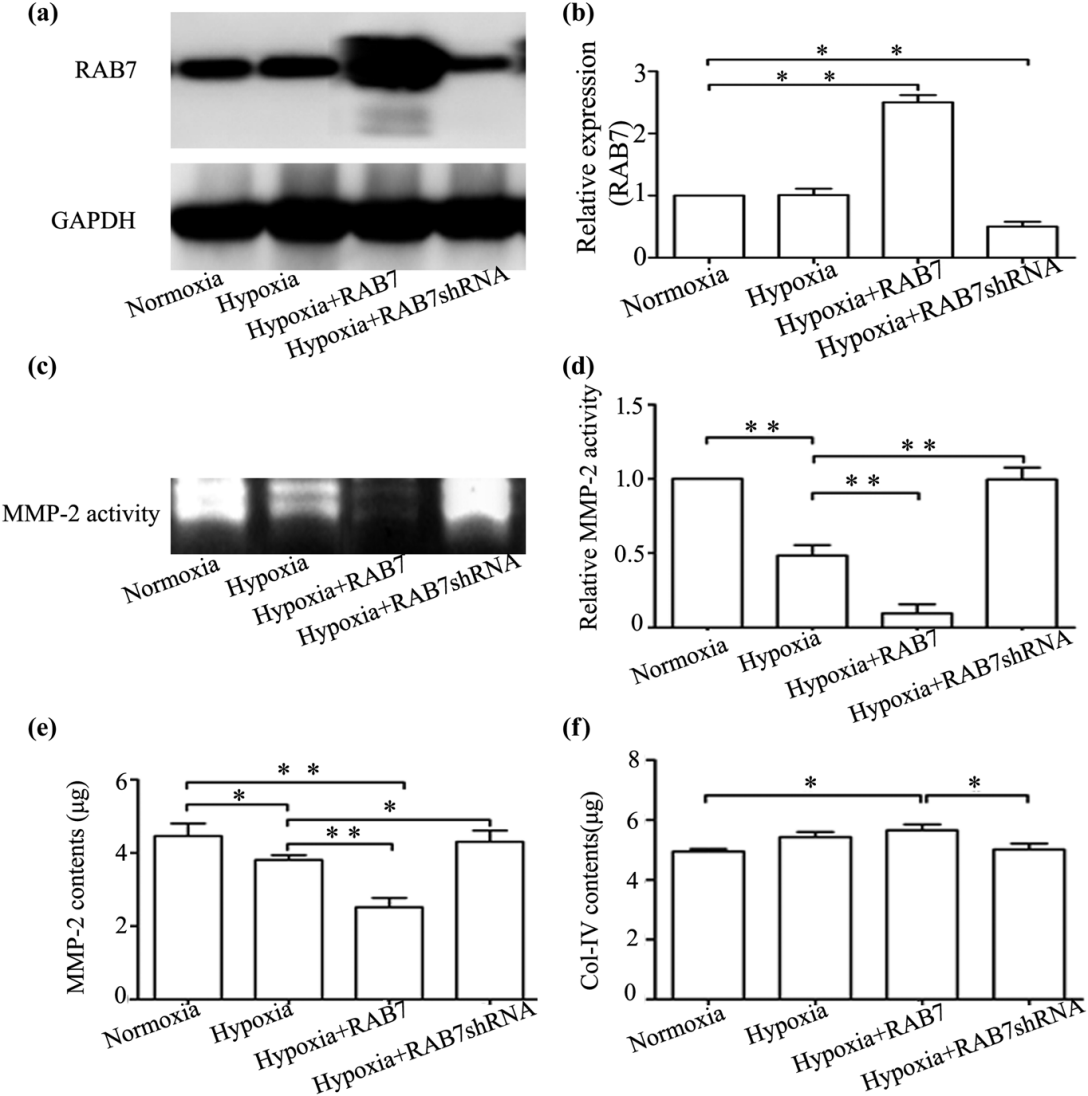
Effect of up- or downregulated RAB7 on MMP-2 activity. (a) Up- or downregulated RAB7 expression in HK-2 cells under hypoxia or normoxia was detected by western blot. The expression of RAB7 was significantly upregulated, and the expression of RAB7 was significantly downregulated compared to the control group. (b) Expression of RAB7 relative to that of GAPDH. (c) MMP-2 activity in culture media was evaluated in HK-2 cells with up- or downregulated RAB7 expression under hypoxia or normoxia by gel zymography, respectively. MMP-2 activity was significantly higher in the group in which RAB7 was downregulated than in the hypoxia group. (d) Gels were scanned and quantified by densitometry and the relative MMP-2 activity was calculated as a percentage of the relevant control values (assigned values: 1) from arbitrary densitometry units. Data are the mean of three separative experiments. (e) The contents of MMP-2 protein in cell culture supernatants were detected by ELISA, respectively. The results were similar to the change in MMP-2 activity in culture media. (f) The contents of Col-IV protein in cell culture supernatants were detected by ELISA, respectively. The results were reversed for MMP-2 protein in the culture media. Error bars represented mean ± SD, and the results were analyzed by unpaired with Student’s *t*-test, *p < 0.05, **p < 0.01.

MMP-2 activity was detected in the following four groups by performing gelatin zymography: (i) Normoxia, (ii) hypoxia, (iii) hypoxia + RAB7 overexpression, and (iv) hypoxia + RAB7 knockdown (Figure 4a and b). MMP-2 activity in the hypoxia group was significantly lower than in the normoxia group (*P* < 0.05). RAB7 overexpression significantly reduced MMP-2 activity under hypoxic conditions when compared with the hypoxia group (*P* < 0.05). In addition, MMP-2 activity was significantly higher in the hypoxia + RAB7 knockdown group than in the hypoxia group (Figure 4c and d).

### 3.4 RAB7 overexpression and knockdown alter the levels of MMP-2 and Col-IV proteins in the culture medium of HK-2 cells under hypoxic conditions

MMP-2 protein content in the cell culture medium was determined by performing an ELISA. As shown in Figure 3e, MMP-2 protein level (3.81 ± 0.13 μg/L) had significantly decreased in hypoxia-treated cells compared to normoxic cells (4.46 ± 0.35 μg/L, *P* < 0.05). In RAB7 overexpression cells following hypoxia treatment for 24 h, MMP-2 protein content (2.52 ± 0.26 μg/L) was reduced compared to the hypoxic group. However, in RAB7 knockdown cells, MMP-2 protein content in the culture medium had increased (4.31 ± 0.30 μg/L; Figure 4e; *P* < 0.05). Alterations in the Col-IV protein level displayed the opposite expression pattern when compared with the MMP-2 protein level (Figure 4f).

### 3.5 Cav-1 and Bec-1 knockdown in hypoxia-treated cells

**Figure 5:**
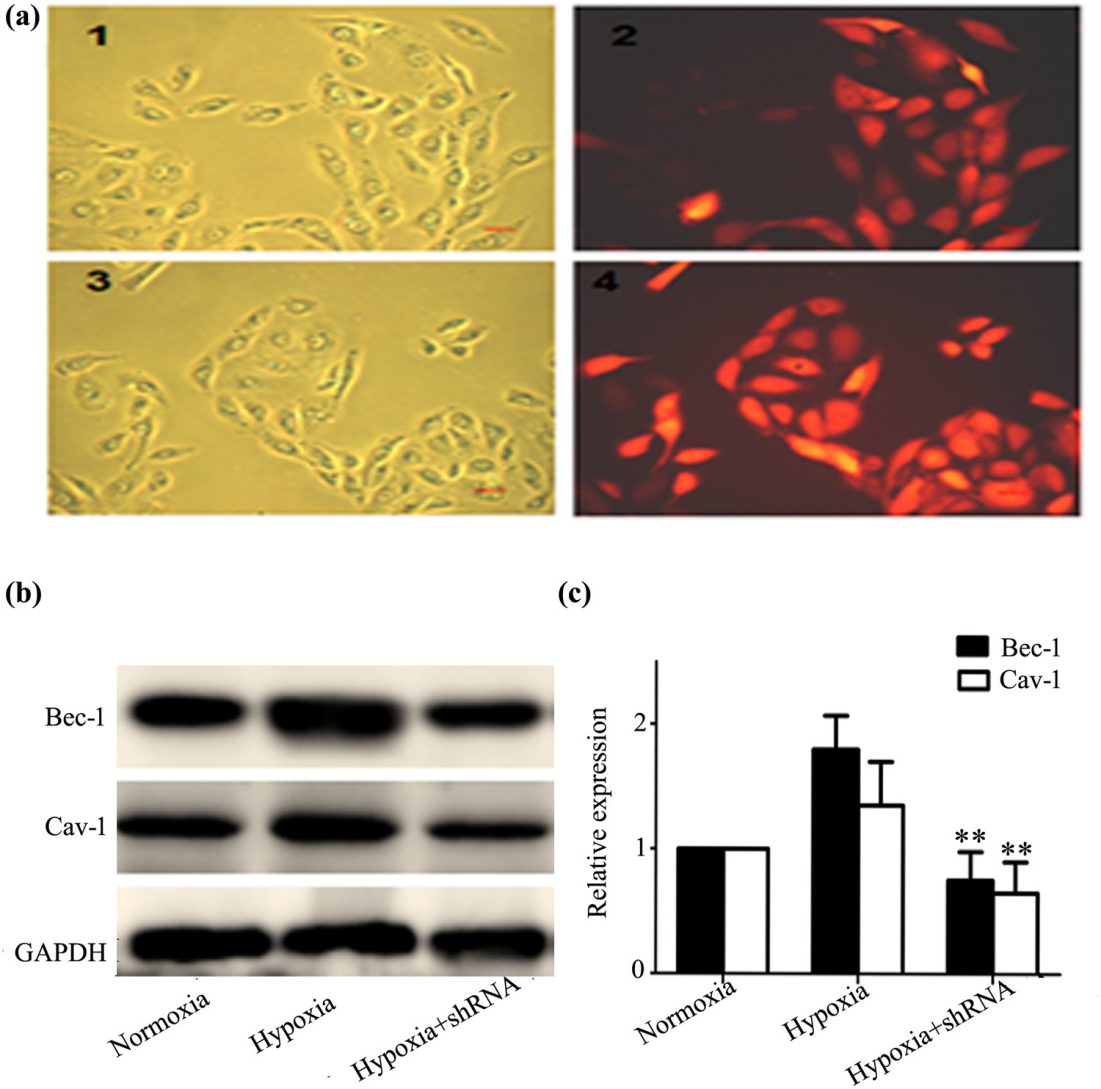
Suppression of Cav-1 by shRNA or Bec-1 by shRNA in hypoxia-treated cells. (a) (1) HK-2 cells (control), (2) Bec-1 shRNA lentiviral vector was infected into HK-2 cells. (3) HK-2 cells (control), (4) Cav-1shRNA lentiviral vector was infected into HK-2 cells. The success rate of infection was almost accounted for 100%. (b) Cav-1 and Bec-1 expression were suppressed in shRNA infected cells. Downregulated Cav-1 and Bec-1 expression in HK-2 cells under hypoxia or normoxia were detected by western blot. Cav-1 and Bec-1 expression levels were suppressed in protein levels when compared with the non-shRNA group in hypoxia. (c) Expression levels of Cav-1 and Bec-1 relative to that of GAPDH. Error bars represented mean ± SD, and the results were analyzed by unpaired with Student’s *t*-test, *p < 0.05, **p < 0.01.

To investigate the roles of Cav-1 and Bec-1 in HK-2 cells, a stable HK-2 cell line (Cav-1 shRNA or Bec-1 shRNA) in which Cav-1 or Bec-1 protein expression levels were knocked down compared to the non-shRNA group under hypoxic conditions was established (Figure 5a–c).

### 3.6 Effect of Bec-1 or Cav-1 knockdown on MMP-2 activity in the culture medium of hypoxia-treated cells

**Figure 6:**
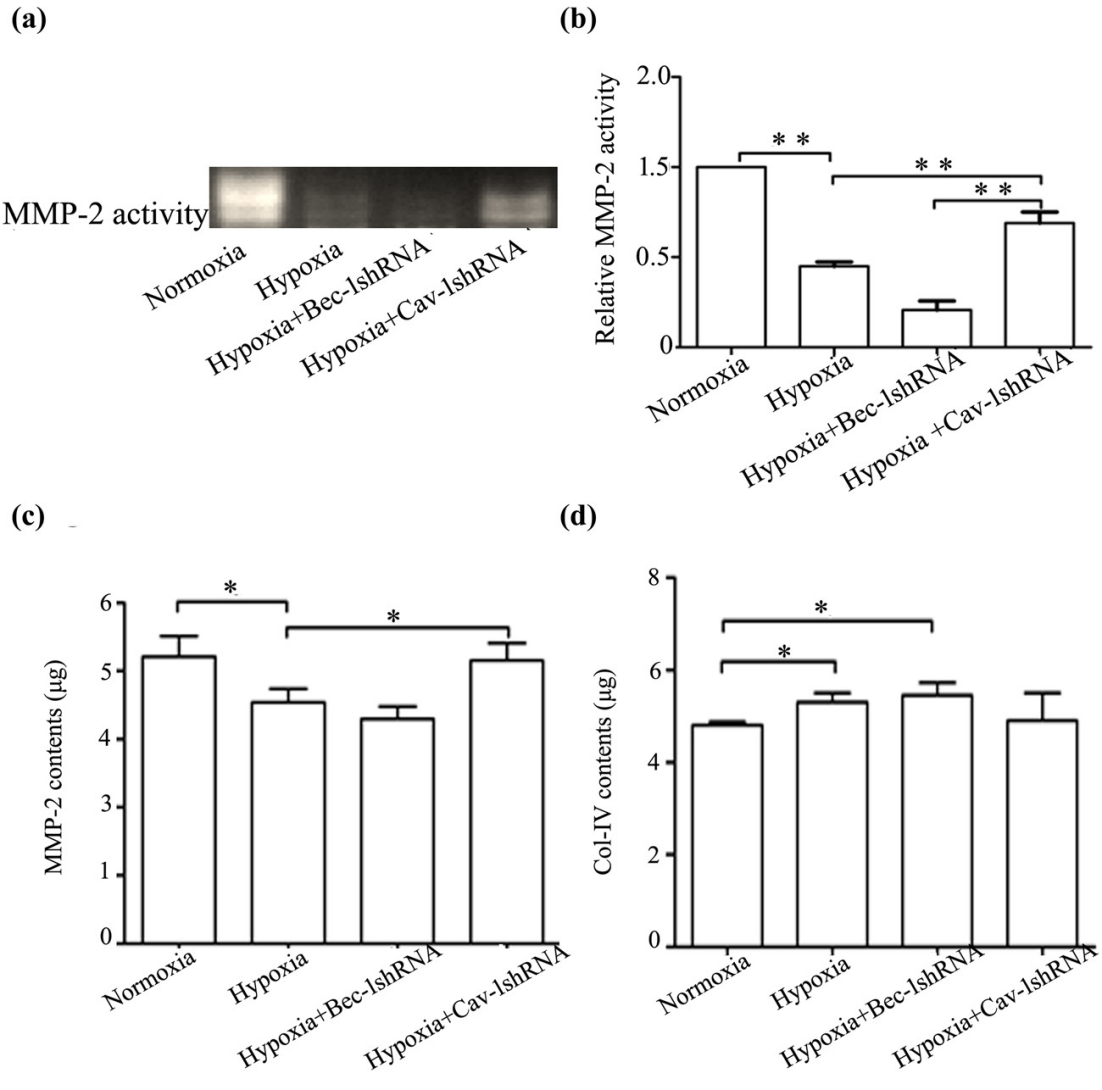
Effect on MMP-2 activity in culture media by Bec-1shRNA or Cav-1shRNA. (a) MMP-2 activity was evaluated by gel zymography in the culture media of hypoxia-treated HK-2 cells. MMP-2 activity was significantly higher in the group in which Cav-1 was downregulated than that in the hypoxia group. (b) Gels were scanned and quantified by densitometry and the relative MMP-2 activity was calculated as a percentage of the relevant control values (assigned values: 1) from arbitrary densitometry units. The data are the mean of the three separative experiments. (c) The contents of MMP-2 protein in cell culture supernatants were detected by ELISA. The results were similar to the change of MMP-2 activity in culture media. (d) The contents of Col-IV protein in cell culture supernatants were detected by ELISA. The results were reversed for MMP-2 protein in the culture media. Error bars represented mean ± SD, and the results were analyzed by unpaired with Student’s *t*-test, *p < 0.05, **p < 0.01.

To determine the effects of Bec-1 or Cav-1 on MMP-2 activity in renal tubular epithelial cells under hypoxic conditions, the normoxia, hypoxia, hypoxia + Bec-1 shRNA, and hypoxia + Cav-1 shRNA groups were assessed via gelatin zymography. Alteration in MMP-2 activity was observed in the cell culture media of the Cav-1 shRNA group (Figure 6a and b). MMP-2 activity in the hypoxia + Bec-1 shRNA group was lower than that in the hypoxia group. In contrast, the MMP-2 activity in the hypoxia + Cav-1 shRNA group was significantly higher than in the hypoxia group (*P* < 0.05). The results indicated that in the absence of oxygen, Bec-1 and Cav-1 knockdown resulted in altered MMP-2 activity between the two groups.

### 3.7 Effect of Bec-1 and Cav-1 knockdown on MMP-2 and Col-IV protein levels in the culture medium of hypoxia-treated cells

The ELISA results showed that the concentration of the MMP-2 protein in the hypoxia + Bec-1 shRNA group was 3.30 ± 0.18 μg/L, whereas the concentration of the MMP-2 protein in the hypoxia + Cav-1 shRNA group was 4.25 ± 0.29 μg/L, which was significantly higher than that of the hypoxia group (3.64 ± 0.25 μg/L). Alteration in Col-IV protein concentration in the cell culture medium displayed the opposite pattern when compared with MMP-2 protein concentration (Figure 6c and d). Moreover, the alterations in the MMP-2 and Col-IV protein content in the culture media reflected the alteration in MMP-2 activity in the culture media.

## 4 Discussion

Renal hypoxia is one of the pathophysiological factors involved in the transition of acute kidney injury to CKD [26]. Previous studies have demonstrated that hypoxia may affect the cellular components of renal tubules, which maintain normal metabolism and function properly, therefore, renal proximal tubule cells are the primary target of hypoxic damage [27]. Autophagy has a close and complex relationship with hypoxia, and serves a dual role in hypoxia-induced cell damage [13,14]. In addition to autophagy, hypoxia can also induce endocytosis [15]. Hypoxia leads to the thickening of the basement membrane and decreased MMP-2 activity [4], but the underlying mechanism remains rarely clear. Our previous study found the effects of autophagy and endocytosis on the activity of MMP-2 in human renal proximal tubular cells under hypoxic conditions. Specific inhibitors of autophagy and endocytosis (3-MA and Filipin, respectively) were used, and the results indicated that autophagy and endocytosis displayed different effects on MMP-2 activity in hypoxia-treated HK-2 cells. Collectively, the aforementioned results suggested that autophagy and endocytosis alter the activity of MMP-2 under hypoxic conditions. However, the underlying mechanism is not completely understood [12,23].

This study, which is an important supplement to previous studies, focused on investigating the effect of RAB7 on MMP-2 activity at the gene level. RAB7 is an effective multifunctional regulatory factor for autophagy and endocytosis [19]. In addition, the relationship between autophagy, endocytosis, and MMP-2 activity was also investigated by analyzing the key genes related to autophagy and endocytosis. First, the expression of RAB7 in human renal biopsy specimens and alteration in the RAB7 expression level in hypoxia-treated GFP-RAB7-overexpression HK-2 cells were assessed. To investigate the effect of RAB7 on MMP-2 activity, RAB7 was overexpressed and knocked down in HK-2 cells under hypoxic conditions. The results demonstrated that RAB7 knockdown significantly increased MMP-2 activity. In contrast, the results for RAB7 overexpression displayed the opposite trend. The results indicated that RAB7 was associated with MMP-2 activity. As RAB7 is an effective multifunctional regulator of autophagy and endocytosis, the potential underlying mechanisms were investigated [19]. The results showed that there is a definite relationship between autophagy, endocytosis, and MMP-2 activity at the molecular level.

To further investigate the possible roles of autophagy and endocytosis in HK-2 cells under hypoxic conditions, shRNA technology was used to reduce the expression levels of Cav-1 and Bec-1 genes in HK-2 cells under hypoxic hypoxia conditions. The Cav-1 shRNA-treated group displayed increased MMP-2 activity, whereas the Bec-1 shRNA group displayed decreased MMP-2 activity compared to the control group. The ELISA results for MMP-2 and Col-IV protein levels in the culture medium were consistent with the gel zymography results. The results of this study were also consistent with previous results. In this study, unlike our previous reported study, we compared the Cav-1 gene with the Bec-1 gene [12,23]. As Bec-1 and Cav-1 are the key genes for autophagy and endocytosis, respectively, autophagy and endocytosis may affect the activity of MMP-2. Some studies have reported that Cav-1 inhibited MMP-2 activity in heart tissue and Filipin decreased caveolae-mediated endocytosis [28,29]. Previous reports have demonstrated that Cav-1 is responsible for maintaining MMP-2 in a membrane-associated and inhibited configuration [28]. The results of this study further suggested that the expression levels of Cav-1 and Bec-1 influenced MMP-2 activity in HK-2 cells under hypoxic hypoxia conditions, which is an important supplement to previous studies. The relationship between autophagy, endocytosis, and MMP-2 activity was further confirmed. However, *in vivo* studies are needed. In conclusion, the findings of this study suggested that autophagy and endocytosis, RAB7, Cav-1, and Bec-1 influence MMP-2 activity in the kidney tissues of patients with CKD. It also suggests that these factors may be associated with the thickening of the basement membrane and accumulation of ECM in renal diseases.

## Abbreviations

CKD: chronic renal disease
ECM: extracellular matrix
MMP-2: matrix metalloproteinase-2
Col-IV: type IV collagen
